# A Working Memory Deficit among Dyslexic Readers with No Phonological Impairment as Measured Using the N-Back Task: An fNIR Study

**DOI:** 10.1371/journal.pone.0046527

**Published:** 2012-11-13

**Authors:** Itamar Sela, Meltem Izzetoglu, Kurtulus Izzetoglu, Banu Onaral

**Affiliations:** 1 School of Biomedical Engineering, Science & Health Systems, Drexel University, Philadelphia, Pennsylvania, United States of America; 2 The Edmond J. Safra Brain Research Center for the Study of Learning Disabilities, University of Haifa, Haifa, Israel; Bellvitge Biomedical Research Institute-IDIBELL, Spain

## Abstract

Data indicated that dyslexic individuals exhibited difficulties on tasks involving Working Memory (WM). Previous studies have suggested that these deficits stem from impaired processing in the Phonological Loop (PL). The PL impairment was connected to poor phonological processing. However, recent data has pointed to the Central Executive (CE) system as another source of WM deficit in dyslexic readers. This opened a debate whether the WM deficit stems solely from PL or can also be seen as an outcome of poor CE processing. In an attempt to verify this question, the current study compared adult skilled and compensated dyslexic readers with no impairment of phonological skills. The participants’ PL and CE processing were tested by using the fNIR device attached to the frontal lobe and measured the changes in brain oxygen values when performing N-back task. As it was previously suggested, the N = 0 represented PL and N = 1 to 3 represent CE processing. It was hypothesized that dyslexic readers who show non-impaired phonological skills will exhibit deficits mainly in the CE subsystem and to a lesser extent in the PL. Results indicated that the two reading level groups did not differ in their accuracy and reaction times in any of the N-Back conditions. However, the dyslexic readers demonstrated significant lower maximum oxyHb values in the upper left frontal lobe, mainly caused due to a significant lower activity under the N = 1 condition. Significant task effects were found in the medial left hemisphere, and the high medial right hemisphere. In addition, significant correlations between fNIR-features, reading performance and speed of processing were found. The higher oxyHb values, the better reading and speed of processing performance obtained. The results of the current study support the hypothesis that at least for the group of dyslexics with non-impaired PL, WM deficit stems from poor CE activity.

## Introduction

Reading is a complex task which is conducted in the information processing system, and as a result a number of cognitive factors are activated, ranging from low-level sensory to high-level cognitive processes, one of which is Working Memory (WM). WM refers to a limited-capacity memory system that involves temporary storage, processing, maintaining, integrating and manipulating of information [1]. According to the proposed model [2], [3], WM consists of two modality-specific slave systems and the modality-free central executive system. The central executive (CE) is the heart of the model and is responsible for the attentional control of the WM, including focusing, dividing and switching attention [3], [4] as well as for coordinating its subsidiary systems and retrieval of information from long-term memory (LTM) [5]. The CE is primarily associated with increased bilateral activity in the frontal and parietal cortices, including the dorsolateral prefrontal cortex, the bilateral premotor cortex and the lateral and medial superior parietal cortex [6]. This subsystem is aided by two modality-specific slave systems: the phonological loop (PL) and the visuo-spatial sketchpad. The PL is assumed to hold verbal and acoustic information, using two subcomponents: a temporary storage system that holds memory traces over a matter of seconds, during which they decay unless refreshed by the second component, the subvocal rehearsal system. The subvocal rehearsal system maintains and registers information within the store [2], [3]. Data indicated that activation of the PL is primarily associated with increased activity in the left inferior parietal lobe, posterior inferior frontal gyrus (IFG), premotor cortex and the cerebellum [7], [8]. The Visuo-Spatial Sketchpad [3] is used in the temporary storage and manipulation of spatial and visual information, such as remembering location, which has been found to promote activation in the parieto-occipital brain region and speed of objects in space, shapes, colors, and planning of spatial movements that were found to promote activation in the inferotemporal region of the brain [6].

Numerous studies have examined the connection between WM subsystems and reading ability. Data indicated that children and adults with reading disabilities exhibited significant difficulties on tasks involving WM. Most of the studies found that the WM deficits among dyslexic readers stem from impaired processing in the PL [9]. Specifically, it was found that the verbal recall span of the dyslexic readers was significantly lower as compared to skilled readers, and as a result the dyslexic readers made inefficient use of the PL, especially in the way that visual information is translated into its phonological form [10]–[15]. Moreover, data indicated this deficit impairs dyslexic readers’ ability to learn new words during reading [9], [12]. Yet, there remains a debate as to whether the source of WM impairment of dyslexics emerges only from a deficit in the PL [16]. Over the years, a large amount of data indicated that phonological processing is the underlining factor of dyslexia [17]; however, behavioral data has pointed to the CE as another source of WM deficit in dyslexic readers [12], [18]–[21]. In addition, a recent functional magnetic resonance imaging (fMRI) study was able to differentiate brain activation during the PL and CE performance among 13-year-old dyslexic readers and age-matched controls [16]. While using the N-Back task [7], where lower activation in the left inferior parietal lobe was observed among the dyslexics as compared to the controls in the performance of N-Back condition where N = 0. This brain area is commonly related to phonological processing among dyslexic adult readers [22], [23] and dyslexic children [24] and in line with the phonological deficit hypothesis [25], [26]. However, when the study sample performed the N-Back tasks at Stage 2, lower activation was observed in the dyslexic readers as compared to the controls in the right IFG and middle frontal gyrus (MFG) and bilaterally in the superior parietal lobule. These fronto-parietal regions are commonly associated with executive processes in adults [6], [27] and children [28]. This study suggests that young dyslexics exhibit two sources of WM impairment; one from the PL and the other from the CE subsystems which are characterized by different abilities [16]. Based on these results, it can be argued that dyslexic readers exhibit domain-specific impairment in the PL and a domain-general impairment in the CE. It is clear that more data is needed to support this claim.

In an attempt to verify WM activation among dyslexic as compared to regular readers, and specifically to verify whether the PL is the only source for WM impairment among dyslexic readers, the participants chosen for the current study were university student (young adult) dyslexic readers with deficits that remained after years of remediation and print exposure. However, the participants had good phonological skills that were matched to a control group of skilled readers. It was hypothesized that dyslexic readers with good phonological skills will exhibit mainly deficits in the CE subsystem and to a lesser extent in the PL. To verify this hypothesis, in the current study we employed N-Back WM task with Stages 0 to 3 [7] while measuring brain activity using Functional Near-Infrared Spectroscopy (fNIR). Based on previous knowledge [16], the N-Back at Stage 0 represented activation in the PL and N-back at Stages 1–3 represented activation in the CE. fNIR has been introduced as a new neuroimaging modality with which to conduct functional brain-imaging studies [29]. fNIR technology uses specific wavelengths of light (700–900 nm), introduced at the scalp, to enable the noninvasive measurement of changes in deoxygenated hemoglobin (deoxy-Hb) and oxygenated hemoglobin (oxy-Hb) concentrations in the cortex during brain activation [30]–[33]. This technology allows the design of portable, safe, affordable, noninvasive, and minimally intrusive monitoring systems. These qualities make fNIR suitable for the study of hemodynamic changes due to cognitive and emotional brain activity under many working and educational conditions, as well as in the field [29], [34]. Several types of cognitive brain function have been assessed by using fNIR, including attention, memory and language tasks such as the lexical decision task [29], [34]–[41]. As our participants were matched on phonological skills, here we hypothesized that brain and behavioral activity of dyslexic and control readers would not differ during the N-Back 0 performance but would be different in N-Back Stages 1–3.

## Methods

### Participants

Twenty-three young-adult skilled readers (age 25.36±3.23, 12 females and 11 males) and nineteen dyslexic readers (age 25.05±2.10, 9 females and 10 males) were recruited to take part in this study. All were native Hebrew speakers from a middle-class background, right-handed, displayed normal or corrected-to-normal vision in both eyes, and were screened for normal hearing. All participants gave their informed consent prior to inclusion in the study, and all were paid volunteers. The skilled readers were recruited by notices posted on bulletin boards on the University campus. The dyslexic readers were recruited from a large pool of participants through the Student Support Service of the University of Haifa, which assists students with learning disabilities. They were diagnosed as dyslexic during childhood and classified as impaired readers by the Student Support Service (reading score of at least −1.5 STD and below on a normative test) [42]. However, for the current study, only participants that had phonological skills within the normal range were selected (see Table 1). None of the participants had a history of neurological or emotional disorders, or attention deficit as measured by the D2 cancellation test [43] (t_(2,89)_ = 1.39, *p* = *0.123,* for the attention D2 test: X = 8.76±1.45, for the dyslexic readers and X = 8.88±1.23 for the skilled readers). Both groups were matched for nonverbal IQ range as measured by the Raven Standard Progressive Matrices [44] (X = 109±2.13 for the dyslexics and X = 110±1.11 for the skilled readers, t_(2,89)_ = 1.06, *p = *0.585), and by the Verbal Equal Side [45] IQ subtest X = 9.37±1.23 for the dyslexics and X = 8.72±1.31 for the skilled readers (T_(2,89)_ = 1.06, *P* = 0.721). The experiment was approved by the University of Haifa Ethics Committee.

**Table 1:** Mean, (standard deviation) and between groups t-test results of dyslexic and regular readers on the baseline measures.

Parameters	Dyslexic readers (N = 17)	Skilled readers (N = 22)	T (df = 2,37)
Words per minute	56.91 (11.269)	120.38 (15.585)	**16.144** ***
Oral reading of connected text (number of errors)	7.869 (4.888)	.461 (.989)	**7.505** ***
Total oral reading time (sec) (216 words)	120.025 (24.893)	82.080 (23.691)	**5.446** ***
Silent reading comprehension (correct from 17)	13.502 (2.728)	14.442 (2.468)	**.244**
Total silent reading time (sec) (479 words)	239.541 (54.459)	138.585 (40.089)	**7.444** ***
Phonology: phonemic deletion from non-words N = 25	24.003 (.541)	24.011 (.267)	**.433**
Phonology: segmentation, N = 16	14.134 (.678)	14.310 (.321)	**.501**
Phonology standard score	.84 (.61)	.89 (.33)	**.554**
Digit span forward	5.739 (.286)	7.230 (1.478)	**3.774** ***
Digit span backward	4.826 (1.072)	6.153 (1.255)	**3.954** ***
Digit span standard score	9.391 (3.056)	12.389 (3.359)	**3.246** **
Working memory for letters standard score	10.800 (2.820)	12.8077 (3.262)	**2.192** **
Speed factor (SOP) (WISA-3 1994)	97.478 (3.488)	117.478 (3.046)	**3.811** ***
Phonological score (out of 41)	39.782 (1.085)	40.269 (1.250)	**1.445**
RAN letter time	26.066 (4.847)	21.222 (3.042)	**3.644** ***
RAN object Time	41.357 (8.490)	32.783 (5.949)	**4.131** ***

* p<0.05;

** p<0.01;

*** p<0.001.

### Measures

#### Decoding Accuracy and Reading Rate

1. One minute tests for words and pseudowords [46]. A list of words (N = 168) and a list of pseudowords (N = 86) were given to the participants. The participants were asked to read each list as fast and accurately as possible in one minute each. The number of correct stimuli read in each list was measured.
2. Oral reading of context (216 words, [42]). Decoding errors and per-letter reading time were measured.
3. Silent reading comprehension of context (476 words, [42]). Reading time and number of correct answers (N = 17) were obtained.

#### Working Memory (WM) Measures

1. Digit Span Forward [45]. The subtest examines accuracy when recalling digits forward (repeating the digits in the order presented).
2. Digit Span Backward [45]. Accuracy is examined accuracy when digits are recalled backward (repeating them in reverse order).
3. Working memory for syllables [47]. The version of the WM letters tests was similar in its construction as the Digit Span [45] but instated of numbers this version uses Hebrew short syllables.

#### Phonological Awareness [42]

1. Phonemic Deletion. This measure contained 25 non-words. The examiner read a word and a specific consonant, and the participant was required to repeat the word without that consonant. Accuracy (the number of correct answers) and the total response time were measured.
2. Segmentation Test. This measure contained 16 non-words. The examiner read each non-word and the participant was required to segment the word into its basic phonological sounds (consonants and vowels) as fast as possible. Time and accuracy were measured.
3. Speed of Processing (SOP) factors. Speed of information processing index comprised of a combined score of two measures: Symbol Search and Coding [45].

#### Experimental protocol: N-Back test

We have utilized N-back test as the experimental protocol for the assessment of WM through the use of hemodynamic measures obtained from FNIR recording. The N-back test is a sequential letter task in which stimuli are single Hebrew consonants presented in a pseudo-random sequence on a computer screen, with a neutral gray background. The task is parameterized by incrementing the WM load from 0 to 3 items. Under the ‘N = 0’ condition the participants were asked to press ‘1’ if the current Hebrew letter is ‘
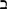
’ (the equivalence to ‘B’ in English), or ‘2’ otherwise. Under the N = 1, N = 2, and N = 3 conditions, the participants were asked to press ‘1’ if the current letter is the same letter as the one appeared N letters before (where N is an integer between 1 and 3) or ‘2’ otherwise for the non-target. The task was presented in four blocks; each block was set to a specific value of N (between 0 and 3). To prevent negative fatigue effect the task protocol was designed to include one block per condition. In addition, in order to maximize participant’s understanding of the task demands the conditions were presented in an incremental order (from 0 to 3). Each block consisted of 30 trials, 10 of them were target trials and the other 20 were non-target trials. In each trial a letter appeared for 400 ms at the center of the screen. The between-trials time interval was set to 4000 ms. The total time length of a given block was 120 seconds. 15 seconds of resting was set prior to each block.

### Apparatus

**Figure 1:**
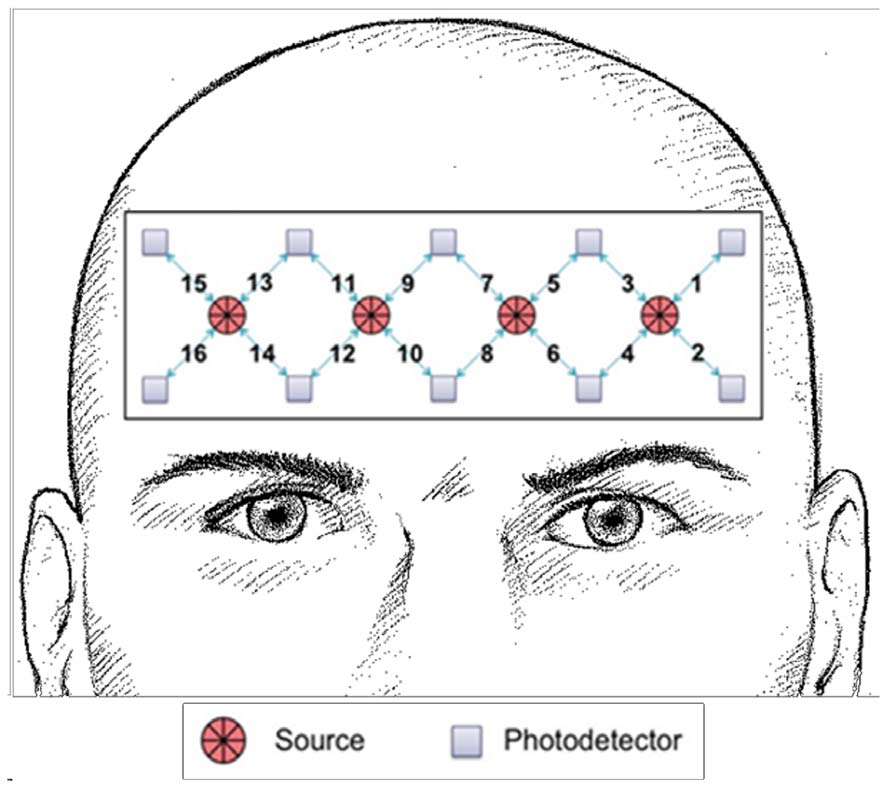
The fNIR Device model 1000. The fNIR head probe was applied to the participant’s forehead. The distribution of four light sources and 10 light detectors resulted in 16 channels.

Two computers and the fNIR device were used in this study:

1. The first computer was used to present the N-Back task stimuli (applied using ePrime software- Psychology Software Tools, Inc. http://www.pstnet.com) and to collect participants’ manual reactions and reaction times.
2. The second computer was used to host the fNIR system (fNIR Devices LLC; http://www.fnirdevieces.com). The fNIR device used in this study was composed of two main parts: a head piece holding the light sources and detectors, and a control box for data acquisition with a sampling rate of 2 Hz. The flexible fNIR sensor consists of four light sources and ten detectors designed to image cortical areas underlying the forehead (dorsolateral and inferior frontal cortices). With a fixed source-detector separation of 2.5 cm, this configuration results in a total of 16 voxels (Figure 1). The control box was connected to the computer for data collection and storage which were utilized by the COBI studio software (Drexel University). In order to synchronize the two computers, a COM cable was used to send online event triggers from the ePrime software to the COBI studio software. Matlab software (Version 2010a, The Mathworks, Natick, MA) was used for the signal processing and to prepare data for statistical analysis which was performed using IBM SPSS (Version 18, IBM SPSS Inc., Chicago, IL).

### Experimental Procedure

Behavioral measures were assessed following an explanation of the experimental procedure. After phonological skills, reading, memory, naming and speed abilities were verified, the selected participants were seated in a sound attenuated room on an adjustable chair facing the computer screen. Then the fNIR sensor was placed on the participants which required about 10 minutes of preparation. Prior to data collection, participants were instructed to respond immediately after the presentation of each stimulus.

The reaction time was defined as the time starting from the stimulus onset until participant’s reaction is received. All trials’ reactions and reaction times were registered to a log file. For each participant, for each block, a mean reaction time and accuracy were computed separately for target and non-target stimulus types.

### fNIR Data Processing and Feature Extraction

The fNIR raw light intensity recorded data was first cleaned from heart pulsation and respiration signals and high frequency noise using a finite impulse response low-pass filter with a cut-off frequency at 0.14 Hz. Then, a ten second time interval that was recorded prior to the beginning of the task was applied to the modified Beer-Lambert law (MBLL) [39] which was used to convert the data to relative changes in hemodynamic responses in terms of oxygenated (OxyHb) and deoxygenated hemoglobin (DeoxyHb). Blood Volume (BL) was computed by summing the OxyHb and the DeoxyHb. Following that, the three hemodynamic response signals were segmented by blocks. For each of the blocks and for each of the three signals, a baseline adjustment was applied by subtracting the mean value of the signal at the 15 second time interval prior to the beginning of the block. Finally, for each block, fNIR-features were extracted for each of the hemodynamic response signals: the minimum, maximum, mean and standard deviation values, as well as the times to reach minimum and maximum value (minimum and maximum times, respectively). Noisy blocks, which mainly occurred due to movement artifacts, were excluded from the statistical analysis.

### Statistical Analysis

A series of repeated measures Analysis Of Variance (rmANOVA) tests were conducted in order to verify group (dyslexic X skilled readers) and task (N = 0,1,2,3) differences in the research parameters. Only correct answers and correct answer reaction times were incorporated into the behavioral measurement statistical analysis. Greenhouse-Geisser correction for non-sphericity was applied where appropriate. A series of between-groups t-tests that was employed to each of the conditions separately followed the different fNIR-feature analyses in which a significant group effect was obtained, in order to identify the difference source.

## Results

The results of the dyslexic and skilled readers on phonological, reading, working memory, naming and SOP measures appears in Table 1. Except for the phonological measures and reading comprehension, the dyslexic readers achieved significantly lower scores in all the other parameters compared to the skilled readers.

### The N-Back Test Behavioral Data Results

#### Accuracy

**Figure 2:**
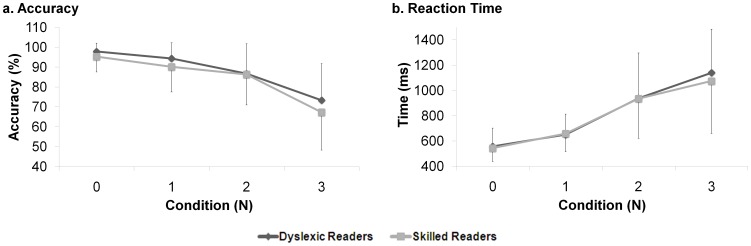
The N-Back Behavioral Results. The dyslexic (dark line) and skilled (light line) readers’ mean (a) accuracy and (b) reaction time under each of the N-Back task conditions. Error bars represent group’s standard deviation.

The comparison between the two reading-level groups revealed that both groups showed a significant N-back effect (F_(3,120)_ = 34.101, p<0.001), thus, the number of correct answers per block decreased as task demand increased (Figure 2a). However, the results also indicated no significant group differences (F_(1,40)_ = 1.90, p = 0.176) in terms of accuracy. Both groups showed higher performance when the N-value was set to ‘0’ (the groups’ accuracy mean values were 95% and 98% for the dyslexic and skilled readers, respectively). Both groups’ performance reduced to 67% for the dyslexic readers and 73% for the skilled readers under the N = 3 condition. No group differences were found when the two groups were compared in each of the blocks separately (t_(26.638)_ = −1.296, p = 0.206; t_(29.031)_ = −1.268, p = 0.215; t_(40)_ = −0.085, p = 0.933; t_(40)_ = −1.041, p = 0.304, for conditions 0–3, respectively).

#### Reaction time

Similar to the results of the accuracy, the analysis of reaction time revealed a significant task effect (F_(3,120)_ = 62.891, p<0.001), together with no group differences (F_(1,33)_ = 0.082, p = 0.776) (Figure 2b.). Both groups showed a relatively fast response under the N = 0 condition (544 ms and 557 ms for the dyslexic and skilled readers, respectively). Both groups’ mean reaction time increased as the task demands increased and reached the values of 1075 ms for the dyslexic readers and 1140 ms for the skilled readers under the N = 3 condition. No between-groups differences were found when the groups’ mean reaction time was compared within each condition separately (t_(40)_ = −0.333, p = 0.741; t_(40)_ = 0.158, p = 0.875; t_(40)_ = −0.018, p = 0.986; t_(40)_ = −0.558, p = 0.580, for conditions 0–3, respectively).

### The N-Back Test fNIR-feature Data Results

The first phase of the fNIR analysis was to investigate which of the 16 channels showed sensitivity to group differences and task demands. The second phase of the analysis was, once these channels were found, to compare the groups in each of the four conditions separately and between each of two successive conditions, in order to investigate whether the between-groups difference and the task effect were stemmed from the phonological loop or the central executive loop.

**Figure 3:**
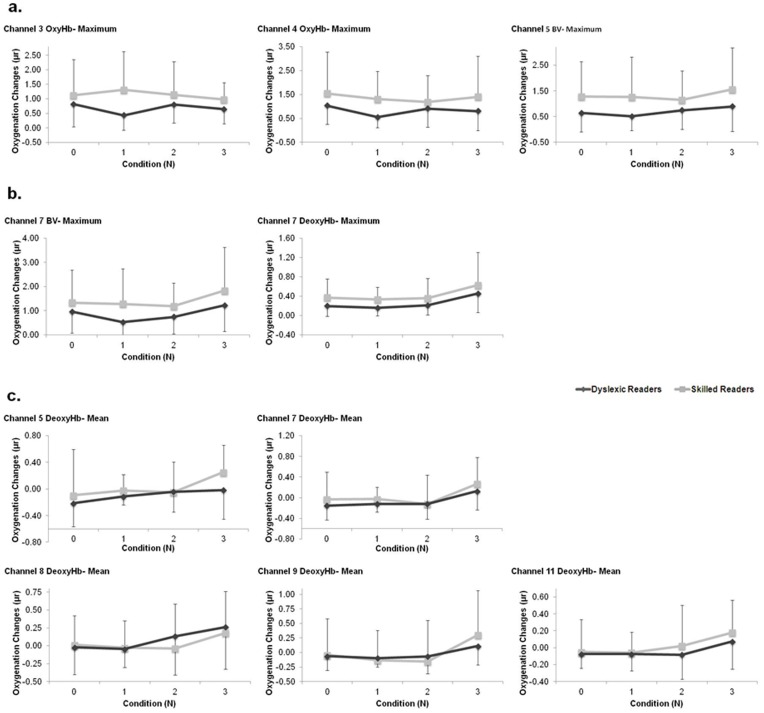
Significant fNIR-features of the dyslexic (dark line) and skilled (light line) readers. a) fNIR-features that showed significant between-group differences. b). fNIR-features that showed both significant group and task effects. c) fNIR-features that showed significant task. Error bars represent group’s standard deviation.

Table 2 summarizes the significant outcomes of the rmANOVA on different hemodynamic signals (OxyHb, DeoxyHb, and BV), channels (1–16), and fNIR-features (Figure 3). Examination of Table 2 revealed that significant between-group differences were found in channels 3, 4, 5, and 7 (Table 2a, and 2b). These channels are located in the left hemisphere, mostly in the higher part (Figure 1). The common fNIR-feature to all these significant comparisons was the Maximum value of the signal during the performance of each of the four task conditions. In all comparisons, the Maximum value of the fNIR signal was higher among the skilled readers as compared to the dyslexic readers (Figure 3a and 3b).

**Table 2:** Significant fNIR-feature based ANOVA results.

	Signal	Feature	Channel	Group	Task
a.	OxyHb	Maximum	3	F_(1,38)_ = 6.689, p = 0.014	NS
	OxyHb	Maximum	4	F_(1,38)_ = 4.777, p = 0.035	NS
	BV	Maximum	5	F_(1,38)_ = 4.945, p = 0.032	NS
b.	BV	Maximum	7	F_(1,38)_ = 4.813, p = 0.034	F_(3,114)_ = 2.661, p = 0.050
	DeoxyHB	Maximum	7	F_(1,38)_ = 5.489, p = 0.024	F_(2.22,84.96)_ = 5.641, p = 0.004
c.	DeoxyHB	Mean	5	NS	F_(1.90,72.52)_ = 3.606, p = 0.034
	DeoxyHB	Mean	7	NS	F_(2.27,86.26)_ = 6.732, p = 0.001
	DeoxyHB	Mean	8	NS	F_(3,114)_ = 3.583, p = 0.016
	DeoxyHB	Mean	9	NS	F_(1.77,67.42)_ = 5.769, p = 0.007
	DeoxyHB	Mean	11	NS	F_(2.63,89.94)_ = 3.615, p = 0.024

a) fNIR-features that showed significant between-group differences.

b) fNIR-features that showed both significant group and task effects.

c) fNIR-features that showed significant task effect. (NS: non-significant).

A series of between-groups t-test comparisons that was applied to each of the four conditions separately in each of the fNIR-features that showed a significant group effect (Table 2a and 2b) revealed a significant difference under the N = 1 condition only (Table 3). Thus, no significant between-groups differences were found under the N = 0, N = 2, or N = 3 conditions (Table 3).

**Table 3:** Between-groups t-test comparison results in the four conditions in the fNIR-features that showed a significant group effect (Table 2a and 2b).

Signal	Feature	Channel	Condition	T
OxyHB	Maximum	3	0	t_(39)_ = −0.924, p = 0.361
			1	t_(29.7632)_ = −2.907, p = 0.006***
			2	t_(38)_ = −1.114, p = 0.272
			3	t_(38)_ = −1.881, p = 0.067
OxyHb	Maximum	4	0	t_(32.034)_ = −1.252, p = 0.219
			1	t_(29.603)_ = −2.8, p = 0.008***
			2	t_(38)_ = −0.874, p = 0.387
			3	t_(38)_ = −1.322, p = 0.193
BV	Maximum	5	0	t_(35.173)_ = −1.95, p = 0.059
			1	t_(28.623)_ = −2.127, p = 0.0421*
			2	t_(38)_ = −1.26, p = 0.215
			3	t_(38)_ = −1.486, p = 0.145
BV	Maximum	7	0	t_(39)_ = −1.035, p = 0.306
			1	t_(28.438)_ = −2.334, p = 0.0268*
			2	t_(38)_ = −1.624, p = 0.112
			3	t_(38)_ = −1.248, p = 0.219
DeoxyHb	Maximum	7	0	t_(39)_ = −1.701, p = 0.096
			1	t_(39)_ = −2.631, p = 0.012*
			2	t_(33.451)_ = −1.546, p = 0.131
			3	t_(38)_ = −0.949, p = 0.348

* p<0.05;

** p<0.01;

*** p<0.001.

The task’s increasing demand for WM had a significant effect on both groups’ fNIR signals (Table 2b and 2c, Figure 3b and 3c). Specifically, evidence for a significant task effect was found in Channels 5, 7, and 8 (medial left hemisphere) and Channels 9 and 11, located at the medial high right hemisphere (Figure 1). Two fNIR-feature types showed sensitivity to task performance. The DeoxyHb signal Mean values of channels 5, 7, 8, 9, and 11 significantly increased as the load on WM increased (Table 2c., Figure 3c). Deeper examination of the results revealed that the task effect was mainly a result of an increase in the DeoxyHb Mean value under condition N = 3. Thus, there were no significant differences between conditions N = 0, N = 1 and N = 2, in any of Channels 5, 7, 8, 9 and 11 (F_(1.59,60.68)_ = 1.316, p = 0.272; F_(1.97, 74.73)_ = 0.246, p = 0.779; F_(1.86, 70.73)_ = 0.618, p = 0.531; F_(1.97, 75.1)_ = 0.478, p = 0.620; F_(2, 76)_ = 0.211, p = 0.810, respectively). However, a paired t-test comparison between condition N = 2 and N = 3 revealed a significant difference in channels 5, 7, 9 (t_(39)_ = −2.063, p<0.05; t_(39)_ = −3.444, p = 0.001; t_(39)_ = −2.952, p<0.01, respectively). In channels 8 and 11, the increased value of the DeoxyHb Mean signal was more gradient, thus, a significant difference was found between conditions N = 1 and N = 3 (t_(39)_ = −2.943, p<0.01; t_(39)_ = −3.309, p<0.01, for channels 8 and 11, respectively), (Figure 3c).

To summarize, fNIR data showed significant effects for both groups and task. These effects are found mostly in the medial left hemisphere, and the high medial right hemisphere. The Maximum fNIR-feature was found to reflect an evidence for between-groups difference. Task effect was mainly reflected in the mean value of the DeoxyHb signal but also in the Maximum value of the BV and DeoxyHb signals in channel 7.

Correlation: In an attempt to verify the relationships between the fNIR parameters and the reading measures, a series of Pearson correlations was conducted. Significant moderate correlations between the number of correct words decoded in a minute and Maximum oxyHB in Channels 3, 4 as well as with the Maximum Blood Volume in channel 5 when performing N = 1 (R = .347 p<.028, R = .410 p<.009, and R = .341 p<.032, for Channels 3, 4, and 5, respectively) were found. The more correct number of words per minute read the higher the amount of Oxygen obtained. Furthermore, significant correlations were also found between the Maximum oxyHb in Channel 4 during the condition of N = 1 and SOP measure (R = −.448 p<.001) and Rapid Automatized Naming (RAN) letter time (R = −.424 p<.007). The lower the SOP score and the longer the performance time for RAN letters, the more oxygen obtained in these channels.

## Discussion

The current study aimed to verify whether WM deficits of dyslexic readers are solely related to impairment in the phonological loop (PL) or whether additional deficits also exist in their central executive system (CE). It was hypothesized that adult compensated dyslexic university students with good phonological skills would exhibit a deficit in the CE and not in the PL. Based on previous studies [16], by using the N-Back task we related the phonological loop activation to N = 0 and the activation of the CE to the N = 1, N = 2 and N = 3 conditions. Furthermore, studies indicated that WM activation, among others, occurs in the frontal lobe [6], [16]. Accordingly, in the current study we employed fNIR imaging technology which can provide measurements from the participants’ frontal lobe.

Although a limited Short Term Memory capacity was observed among the dyslexics as compared to the skilled readers group in the Digit Span [45] baseline test (Table 1), no significant differences were found between the two reading-level groups either in behavioral accuracy or in reaction time when performing the N-Back experimental test. This discrepancy in results may be taken as evidence that even though both Digit span and N-back are considered to be WM tasks they reflect different memory mechanisms [48]. Thus, while digits span measures the size of a memory buffer [45], N-back requires an ongoing buffer update [49]. As opposed to recent research [16] who studied phonologically impaired young dyslexics adolescents, our dyslexic participants were adult compensated university students, who fell within the normal range of phonological skills, relatively similar to the skilled readers. Based on the causal contribution of phonological skills to reading and the relationships between WM and the reading process, it is conceivable that good phonological skills might affect not only PL processing but also contribute to the entire WM process. A support for this assumption may be taken from the fact that no between-group differences were found in any of the fNIR features under the conditions of N = 2 and N = 3. Thus, it is probably that the current study’s compensated dyslexic readers do not fall back in their task performance as they enjoy a relatively high-functioning WM buffer update abilities. However, it is clear that future replications of the current study protocol that will employ dyslexic readers with phonological impairment might verify these assumptions.

Nevertheless, as opposed to the behavioral outcomes of the N-Back task, significant between-group and task effects were obtained in the fNIR data. As previously suggested [50], the behavioral measurement such as accuracy and reaction time reflect only the process’s outcome. As such, behavioral measures cannot provide information regarding the stages of activation during cognitive process. However, the neuroimaging measurement seems to help to “divide” the process into its subcomponents and to verify activation in its different stages. As can be seen, our fNIR results showed group and task effects in several of the fNIR-features. Specifically, data indicated that the medial left frontal lobe, mainly at the higher part of the hemisphere, showed sensitivity to group differences in the N = 1 condition. There, fNIR-features indicated significantly lower oxygen among the dyslexic readers as compared to the controls. Although our results did not show significant between-group differences in the N = 1 accuracy, the mean N = 1 accuracy score was lower and standard deviation was higher in the dyslexic readers as compared to the skilled reader (Figure 2a). It is possible that less oxygenation causes lower performance.

Furthermore, if it is accepted that N = 1 to N = 3 can indicate CE processing, it can be suggested that the skilled readers might have showed more effective CE activation, and comparatively the dyslexic readers exhibited lower CE activation under the N = 1 condition. It was claimed [3] that the CE is a modalities-free subsystem. Namely, not only processing in the PL and the visual scratch pad affect processing in the CE but also more general characteristics of cognitive activity. As our data indicated, out of all of our research measures the lower oxygenation in the N = 1 task was found to be related to slow SOP and slower performance in the RAN letter task. In different studies, SOP was found to be also a more domain-general and not only modality-specific factor [51], [52]. Furthermore, slow SOP was suggested to be one of the underlying factors of dyslexia [53]. In addition, in different studies the slower RAN for letters that was found among dyslexic readers was connected among others to slow general SOP [51]. Accordingly, it can be suggested that the low oxygenation exhibited in the CE among the dyslexic readers might be related also to more general slow SOP. However, whether SOP affects the physiological activation of CE subsystem or the CE affects the SOP needs to be study in depth.

In addition, it was suggested that the left frontal lobe is involved in verbal WM with low executive demand [6]. Support for this notion can be found in the current study's N-Back results under the condition of N = 1, as inferior verbal processing has been suggested to characterize dyslexia and between-group differences were found in the left hemisphere (Table 2a and 2b, Figure 3a and 3b). However, it should be noted that the current study’s stimuli were un-pointed Hebrew uppercase letters. One cannot rule out the possibility that although part of the task is letter recognition, i.e. phonological, in order to overcome individual disadvantages, the dyslexic readers bypassed their phonological processing by using visually-based processing strategies therefore it could be that the 0-back condition may not be indicator for PL. This point may be overcome in a future study in which task stimuli will include both pointed and un-pointed upper and lower case letters. Thus, the combination between the different letter versions might force the participants also to use the phonological loop as well.

Furthermore, our study also indicated no between-group differences under the N = 2 and N = 3 conditions in any of the fNIR-features and within-group analysis, as N = 3 was the most difficult task for both groups. Thus, it can be suggested that the increase in task demands was difficult for the two reading level groups resulting in similar results for all parameters. Moreover, it is conceivable that difficult tasks required the involvement of other brain regions not located in the frontal lobe [6]. As it was found in our study, the differences between the task conditions were observed within the two groups in medial left and right parts of the frontal lobe. This might indicate that regardless of the level of reading, when the level of difficulty on the CE increases the activation is less lateralized and spreads in both sides of the brain.

In a recent fMRI study [16] a modified verbal version of the N-Back task was used to compare young dyslexic readers’ WM with their skilled reader peers. The results indicated that among the skilled readers, the left IFG and MFG were activated under the N = 0 condition. However, under the N = 2 condition, activity was found in the right IFG, MFG, and the superior frontal gyrus (SFG) as well. Unlike the skilled readers, the dyslexic readers demonstrated a bilateral activity under the N = 0 condition, specifically in the left and right IFG and MFG, and a significant activity in the left SFG as well as in the right MFG and SFG under the N = 2 condition. Significant between group differences were found in the right IFG, MFG under the N = 0 condition and in the right IFG under the N = 2 condition. Our results support partly the above fMRI N-Back results by showing group differences in the left IFG and MFG that can be consider as more related to brain language areas [54]. However, as opposed to Beneventi et. al [16], the current study showed increase in brain activity in Channels 5, 7, 8, 9, and 11, which may correspond to right and left SFG [29] among both reading level groups as a result of increase in the memory task demands. It is conceivable that the differences between the two studies came as an outcome of the age and the characteristic differences of the two studies samples. Beneventi et. al [16] sample included dyslexic readers children with impaired phonological skills and the current study dyslexic readers were adult university students. They were defined as compensated dyslexic readers. It may be the case that after years of print exposures and remedial teaching the adult dyslexic readers managed to compensate some of their deficits in the WM domain but less in their language domain.

Finally, the results of the current study provide additional evidence for the relevance of fNIR in brain research. However, together with the advantages of the technique, one should be aware of its limitations. With the source detector separation (2.5 cm) used in the device implemented in this study (fNIR Devices, 1000), the penetration depth of the near infrared light in participant's head was limited to the surface of the cortex only [39]. Moreover, the device used in the current study was designed to be applied only on the forehead. Hence brain activity was documented only from the prefrontal cortex [39], [55]. Although working memory tasks highly involved frontal lobe activity [27], [56], the current study’s setup cannot contribute to the understanding of the relationships between the frontal lobe and other brain regions. Furthermore, while interpreting the current study results it is important to note that the N-Back blocks were presented to the participants in a fixed order and without blocks repetition. A further study should take it into consideration. In addition, some recent studies investigated the effects of superficial tissues, mainly the skin and scalp on the changes in hemodynamics in response to cognitive tasks [57]–[60]. These studies suggested that since fNIR measures a volume of tissues underneath the source and detector pair of investigation there is a possibility that changes in the hemodynamics within the skin/scalp layer may be correlated with the task used and can get detected by the fNIR sensor together with the changes within the brain layer. In this study as well there is a possibility that the observed changes are partially coming from the skin/scalp layer. Since in this study the fNIR probe used is not multi-distance it is hard to rule out this possibility. However, the statistically significant differences in certain hemodynamic parameters as measured by the fNIR system are observed only in certain channel locations. This may suggest that the changes detected are most probably coming from the brain since it can be assumed that if there was skin/scalp related changes due to the task it should have been observed in most of the fNIR channels. However, the skin/scalp effect in the task used in this study has to be investigated in the future using a multi-distance probe. In that setting hemodynamic changes in superficial tissues as measured by small source-detector distance pairs (∼1 cm) should be correlated with changes in deeper tissues including the brain layer as measured by larger source-detector pairs (∼2.5 cm as used in this study) in order to fully rule out this effect.
